# Effect of Double-Ovsynch and Presynch-Ovsynch on postpartum ovarian cysts and inactive ovary in high-yielding dairy cows

**DOI:** 10.3389/fvets.2024.1348734

**Published:** 2024-02-01

**Authors:** Ziyuan Li, Shuyi Luan, LongGang Yan, Chengyun Xie, Zhengjie Lian, Mingmao Yang, Minmin Mei, Pengfei Lin, Aihua Wang, Yaping Jin

**Affiliations:** ^1^College of Veterinary Medicine, Northwest A&F University, Yangling, China; ^2^Key Laboratory of Animal Biotechnology, Ministry of Agriculture and Rural Affairs, Northwest A&F University, Yangling, China; ^3^School of Animal Science and Technology, Guangdong Polytechnic of Science and Trade, Qingyuan, China

**Keywords:** Double-Ovsynch, Presynch-Ovsynch, ovarian diseases, ovarian cysts, inactive ovary

## Abstract

**Introduction:**

Optimizing the management of dairy cattle reproduction can reduce postpartum ovarian disease in high-yielding dairy cows and thus enhance ranch economic benefit. The hypothesis of this study was that the Double-Ovsynch (DO) protocol in high-producing dairy cows would result in a lower incidence of follicular cysts but a higher incidence of luteal cysts compared to those undergoing the Presynch-Ovsynch (PS) protocol.

**Methods:**

In this experiment, 384 cows (204 primiparous and 180 multiparous) were allocated to the DO group, which followed the protocol: GnRH-7d-PGF2α-3d-GnRH-7d-Ovsynch-56 h (GnRH-7d-PGF2α-56 h-GnRH-16hTAI), starting on 39 ± 3 days in milk (DIM). Additionally, 359 cows (176 primiparous and 183 multiparous) were assigned to the PS group, which followed the protocol: PGF2α-14d-PGF2α-12d-Ovsynch-56 h, starting on 31 ± 3 DIM. In DO, B-mode ultrasound examinations were conducted 1 day after the GnRH-7d-PGF2α-3d-GnRH protocol to diagnose the presence of ovarian diseases followed by reexamination after 7 days of suspected cases. In PS, B-mode ultrasound examinations were conducted 1 day after the PGF2α-14d-PGF2α protocol to diagnose the presence of ovarian diseases followed by reexamination after 7 days. For all cows confirmed to having ovarian diseases, a second B-mode ultrasound examination was conducted at the time of the second GnRH and timed artificial insemination (TAI). If the ovary showed a normal developing follicle in combination with normal ovulation, the ovarian disease was considered to be cured.

**Results:**

The current study revealed no significant difference in the overall incidence and cure rate of postpartum ovarian diseases between DO and PS (incidence rate: 3.9% vs. 6.7%, cure rate: 50% vs. 41.7%, DO vs. PS). Also, there was no significant difference in the incidence and cure rate of luteal cysts between DO and PS (incidence rate: 2.9% vs. 2.2%, cure rate: 50.0% vs. 50.0%). The incidence of follicular cysts was significantly lower in the DO group than in the PS group (0.8% vs. 2.8%, DO vs. PS, *p* = 0.037), but there was no significant difference in the cure rates (66.7% vs. 50%). The occurrence of inactive ovary was lower in DO compared to PS (0.2% vs. 1.7%, *p* = 0.047). There was no significant difference in the pregnancy rate between the DO and PS groups (48.2% vs. 41.8%), although the DO group had a higher rate. What is different from our assumption is that PS did not effectively reduce the incidence of postpartum luteal cysts.

## Introduction

Postpartum ovarian diseases are the primary cause of reproductive disorders in high-yielding dairy (1, 2). Common production-related ovarian diseases include luteal cysts (3), follicular cysts (4), inactive ovary (5, 6), and persistent corpus luteum (7). The high incidence of postpartum ovarian diseases in high-yielding dairy cows can prolong the waiting period for cows, reduce the first conception rate, and consequently diminish ranch profitability (8). Currently, ovarian diseases are primarily treated with hormone drugs (9), similar to those used in ranch reproductive protocols.

Both luteal and follicular cysts are considered cystic ovarian conditions (4), though their precise definitions remain a subject of debate. According to current diagnostic criteria, a luteal cyst is identified when a luteum body larger than 20 mm in diameter is present on either side of the ovary and persists for more than 7 days (10). A follicular cyst is characterized by the presence of a follicle larger than 20 mm in diameter on either side of the ovary which persists for more than 7 days. During rectal examination using B-mode ultrasound, a cyst is classified as either luteum or follicular if its wall thickness exceeds or is below 3 mm, respectively. Moreover, follicular cysts are characterized by progesterone levels below 1 ng/mL (10, 11). The incidence of ovarian cysts is generally between 2.7 and 30% in the first 15 to 40 days after calving. Previously demonstrated that, in the absence of any other interventions, an increase in milk production of 500 kilograms might result in a 1.5% increase in ovarian cysts incidence (12).

In clinical practice, inactive ovary is known by various terms, including inactive-static ovary (13), ovarian hypofunction (14, 15), and ovarian quiescence (16). Diagnostic criteria for ovarian quiescence include the absence of follicles larger than 7 or 10 mm on both ovaries, the absence of corpus luteum, as well as a duration exceeding 7 days. Moreover, assisted serum testing revealed progesterone concentrations of less than 1 ng/mL (13). Without intervention, the incidence of postpartum cattle disease remains below 8.5% (5).

Currently, ovarian cysts can be treated according to several methods, including manual rupture (17), aspiration (18), and hormone therapy (9, 18); with the latter being the most commonly used. A single injection of prostaglandin F2-alpha (PGF2α) can be used to treat luteal cysts (19), while a single injection of gonadotropin-releasing hormone (GnRH) or human chorionic gonadotropin can be used to treat follicular cysts (9); both with significant therapeutic effects. Post-treatment, there is an estrus rate of 75% and a pregnancy rate of 66% (20). The Ovsynch protocol was used to treat ovarian cysts in cows, but the pregnancy rate after treatment is low (21). Previously, Branko et al. and others used GnRH and equine chorionic gonadotropin (eCG) to treat inactive ovaries. Whereas both were effective treatments, eCG increased the probability of cows laying twinning rate (13). Additionally, although platelet-rich plasma does have therapeutic effects on inactive ovaries (22), it requires ovarian injection which is a more complex administration method and as such is more difficult to use clinically.

In commercial dairy farms, ovarian diseases are rarely diagnosed and treated. Postpartum cows are directly subjected to Double-Ovsynch (DO) and Presynch-Ovsynch (PS) protocol. While hormones used in the synchronization protocol have therapeutic effects on ovarian cysts and inactive ovaries, relevant data is lacking. This study aimed to assess the influence of the DO and PS reproductive protocols on incidence and cure rates of postpartum ovarian cysts and inactive ovary in high-yielding dairy cows. The objective is to provide data for herd reproductive management and to serve as a reference for the treatment and prevention of ovarian diseases.

## Materials and methods

### Animals and farms

A total of 743 postpartum Holstein cows without a history of disease were selected from one commercial dairy farms in Ningxia, China. Cows were raised in barns equipped with sand beds, a feeding lock door, and exercise yard. The cows were fed a mixed total daily ration, mainly silage, which is formulated by professional nutritionists according to the protein, vitamins, and energy required by the cows. The cows were fed three times a day with TMR, which is composed of corn silage, alfalfa, soybean meal, extruded corn, etc. to meet the minimum nutritional needs of the animals. Every day, the commercial ranch management software, Yi Muyun (Yimu Technology Co., Ltd.), was used for dairy cattle management, including the implementation of breeding programs, disease detection, and milk recording. All animal experiment protocols were approved by the Animal Care Committee of the School of Veterinary Medicine, Northwest A&F University and were carried out when the cows were fed and locked in their stalls (Table 1).

**Table 1:** Basic information table for each group of dairy cows.

Item	Calving number	Milk yield	Body condition score
Double-Ovsynch (*n* = 384)	1.85 ± 0.3	53.1 ± 3.4	2.92 ± 0.3
Presynch-Ovsynch (*n* = 359)	1.78 ± 0.4	54.5 ± 5.3	2.87 ± 0.4

### Methods and materials

A total of 743 cows were randomly divided into two groups. In the DO group, the Ovsynch protocol was performed for pre-Ovsynch at 39 days postpartum, PGF2α (Aminobutyl triazol propane sulfonate, Ningbo Sansheng Biotechnology Co., Ltd.) was injected 7 days after the first injection of GnRH (Ningbo Sansheng Biotechnology Co., Ltd.) followed by an additional GnRH injection 3 days after the PGF2α injection. Then, B-ultrasound examination of the ovaries was performed. Seven days later, the Ovsynch-56 TAI protocol was started and B-ultrasound (7.5 MHz Line Array Probe, IMV Technologies Group) examination of the ovaries was performed when the first injection of GnRH in the Ovsynch-56 TAI protocol was used. In the PS group: Presynch-Ovsynch protocol was used for pre-Ovsynch, the first injection of PGF2α was given at 31 days postpartum followed by a second injection of PGF2α 14 days later. B-ultrasound examination of the ovaries was performed using a rectal probe 24 h later and repeated after 7 days. Ovsynch-56 TAI was started 12 days after completion of pre-Ovsynch. All cows received the Ovsynch-56 TAI protocol consisting of GnRH at 56 ± 3 days in milk (DIM), PGF2α 7 days later, GnRH 56 h after PGF2α, and TAI 12 to 14 h later at 66 ± 3 DIM. Renal B-ultrasound examination of the ovaries was performed 24 h after PGF2α in the Ovsynch-56 TAI protocol, followed by a recheck during TAI.

### Diagnosis and cure criteria of ovarian diseases

**Figure 1:**
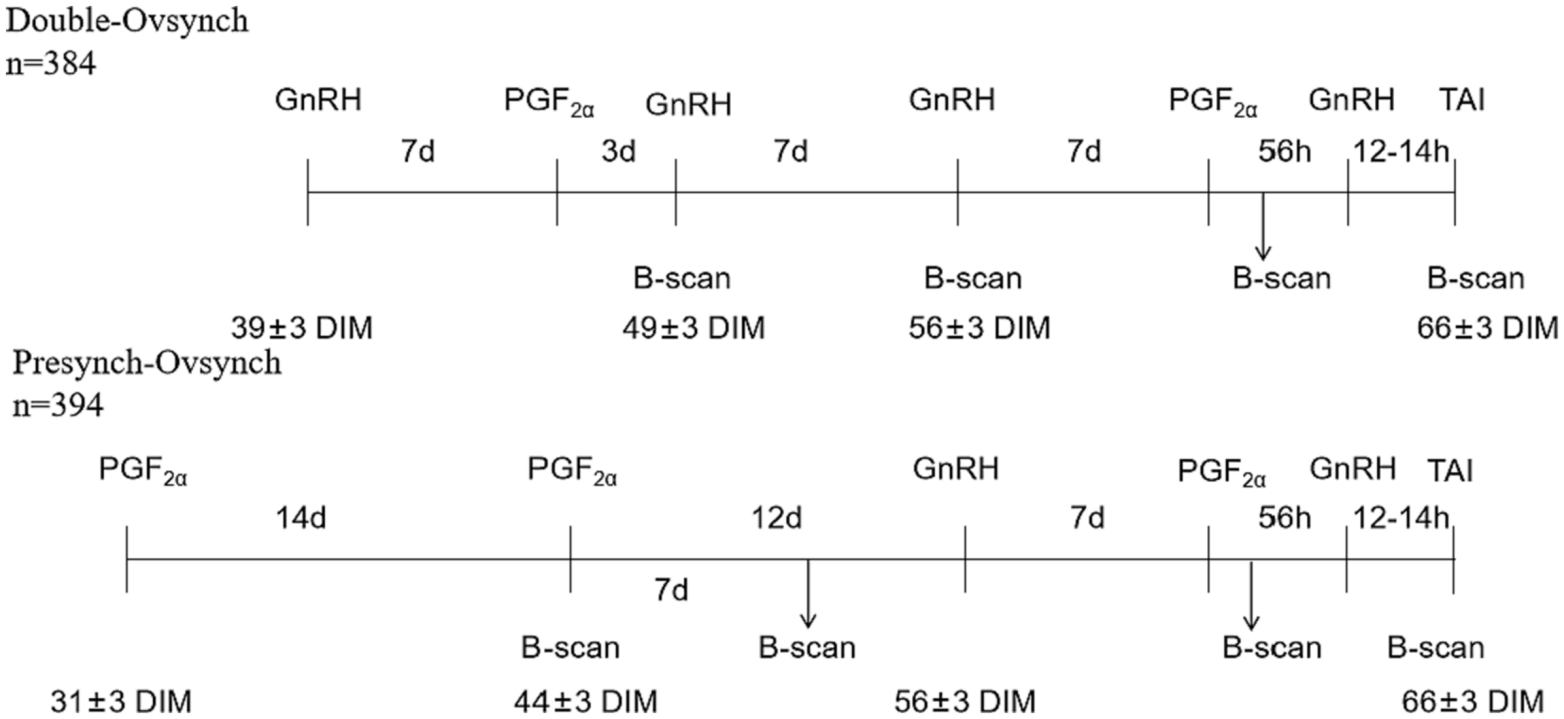
Schematic diagram of hormonal treatments and B-ultrasound in lactating dairy cows (mean ± range of DIM).

**Figure 2:**
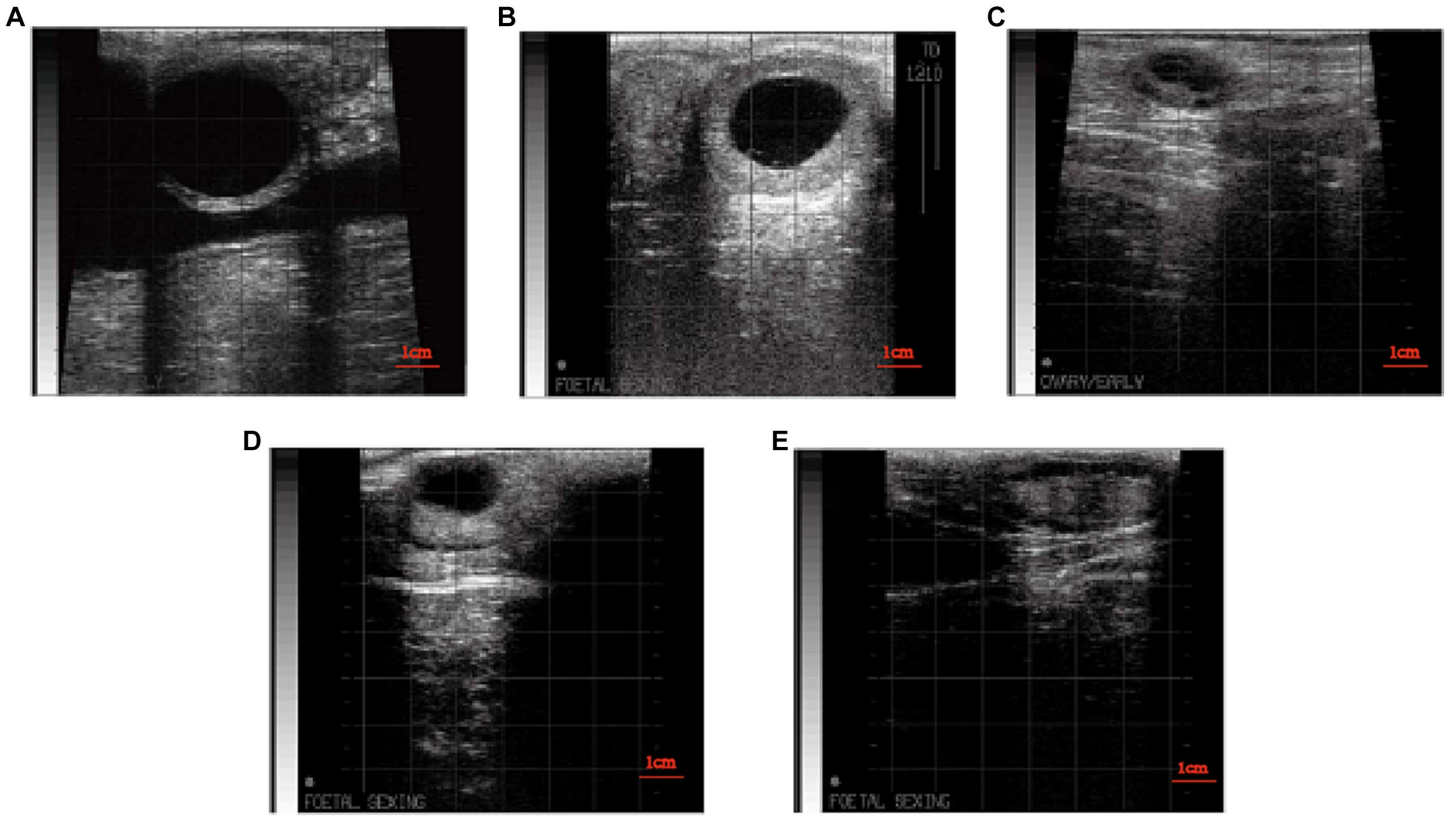
**(A)** B-mode ultrasound image follicular cysts. **(B)** B-mode ultrasound image of luteal cysts. **(C)** B-mode ultrasound image of inactive ovaries. **(D)** B-mode ultrasound image of dominant follicle after treatment of ovarian disease. **(E)** B-mode ultrasound image of dominant follicle after ovulation.

The status of both ovaries was examined using B-ultrasound, and the examination time is shown in Figure 1. When there is a corpus luteum larger than 20 mm on one or both ovaries, it is diagnosed as a luteal cyst when the re-examination is performed after 7 days (Figure 2B) (10). When there were follicles larger than 20 mm on one or both ovaries, the large follicles still existed and were larger than 20 mm after 7 days (Figure 2A) (10). Serum progesterone was measured by ELISA (Cloud-Clone Crop ELISA Kit for Progesterone (PG) limit of detection: 0.47 ng/mL). Follicular cysts was diagnosed when the concentration of progesterone was less than 1 ng/mL, and corpus luteal cysts was diagnosed when the concentration of progesterone was more than 1 ng/mL. When there are no follicles larger than 7 mm and no corpora lutea on both ovaries, and re-examination after 7 days, the follicles on both ovaries are still smaller than 7 mm and there is no corpus luteum (Figure 2C) (10). Blood was collected from the tail for serum separation, and ELISA was used to measure the progesterone content in the serum. When the progesterone concentration was <1 ng/mL, it was diagnosed as an inactive ovary. Before and after TAI, B-ultrasound examination of the ovaries was performed. If there were no corpora lutea or follicles larger than 20 mm on the ovaries with ovarian cysts, and there were follicles larger than 7 mm on the inactive ovaries that could ovulate normally (Figures 2D,E), then the ovarian disease was considered cured.

### Pregnancy diagnosis

During the 33 days after TAI, rectal ultrasound pregnancy examination was performed using B-ultrasound (7.5 MHz Line Array Probe, IMV Technologies Group). Before that, cows with return of estrus were diagnosed as non-pregnant.

### Compliance with the protocol

A total of 756 clinically healthy cows were selected from one farms. Cows that were culled, sick, or died during the study period (*n* = 8), and cows that did not follow the DO or PS breeding protocol (*n* = 5) were removed from the dataset. Finally, a total of 743 healthy cows were used for analysis in the two farms. Among them, there were 384 cows in the DO group and 359 cows in the PS group.

### Statistical analysis

Following daily recording, the data were transferred to Excel for straightforward classification and conversion into binomial form. Subsequently, SPSS 26.0 (SPSS Inc., Chicago, IL, United States) was used for Pearson chi-square test analysis, with statistical significance set at *p* < 0.05.

## Result

### Incidence of ovarian diseases in the Double-Ovsynch and Presynch-Ovsynch

The occurrence of inactive ovary was significantly lower in DO compared to PS (0.2% vs. 1.7%, *p* = 0.047). The incidence of follicular cysts was significantly lower in the DO group than in the PS group (0.8% vs. 2.8%, DO vs. PS, *p* = 0.037). There was no significant difference in the incidence and cure rate of luteal cysts between DO group and PS group (incidence rate: 2.9% vs. 2.2%, DO vs. PS). An overview of the data is shown in Table 2.

**Table 2:** Effect of Double-Ovsynch or Presynch-Ovsynch on the incidence of postpartum ovarian diseases.

Item	Luteal cysts	Follicular cysts	Inactive ovary	Overall
Double-Ovsynch	2.9%	0.8%	0.2%	3.9%
	(11/384)	(3/384)	(1/384)	(15/384)
Presynch-Ovsynch	2.2%	2.8%^*^	1.7%^*^	6.7%
	(8/359)	(10/359)	(6/359)	(24/359)

*p-values for comparison of Double-Ovsynch versus Presynch-Ovsynch (*p* < 0.05).

### Cure rate of ovarian diseases in the Double-Ovsynch and Presynch-Ovsynch

Compared to PS, DO showed higher cure rates for luteal cysts, follicular cysts, and inactive ovaries; however, the differences failed to reach statistical significance. Also, a similar trend in the overall cure rate of ovarian diseases was observed (Table 3).

**Table 3:** Effect of Double-Ovsynch or Presynch-Ovsynch on the cure of postpartum ovarian diseases.

Item	Luteal cysts	Follicular cysts	Inactive ovary	Overall
Double-Ovsynch	54.5%	66.7%	100.0%	60.0%
	(6/11)	(2/3)	(1/1)	(9/15)
Presynch-Ovsynch	50.0%	50.0%	16.7%	41.7%
	(4/8)	(5/10)	(1/6)	(10/24)

The Pregnancy Rate of the Double-Ovsynch and Presynch-Ovsynch groups.

The pregnancy rate in the DO group was 1.15 times higher than that in the PS group; however, this difference did not reach statistical significance. Following the DO and PS procedures, the pregnancy rate in cured dairy cows with ovarian diseases was higher in the DO group compared to the PS group. However, this difference might be attributed to the small sample size and lack of statistical significance (Table 4).

**Table 4:** The pregnancy rate of the Double-Ovsynch and Presynch-Ovsynch groups.

Item	Pregnancy rate	Pregnancy rate after the cure of ovarian diseases
Double-Ovsynch	48.2%	55.6%
	(185/384)	(5/9)
Presynch-Ovsynch	41.8%	20%
	(150/359)	(2/10)

## Discussion

The hypothesis of this study was that the Double-Ovsynch (DO) protocol in high-producing dairy cows would result in a higher incidence of luteal cysts compared to those undergoing the Presynch-Ovsynch (PS) protocol and incidence of follicular cysts is lower in the DO group than in the PS group. The exact mechanism of ovarian cyst formation is not fully understood, but it is generally accepted that disruption of the hypothalamo-pituitary-gonadal axis, by endogenous and/or exogenous factors, causes cysts formation (23).

In the treatment of luteal cysts, GnRH accelerates the healing time of luteal cysts. Progesterone on the base of the plateau can inhibit the surge of LH but increase the frequency of LH (24). GnRH can increase blood flow to the ovaries (25), and the high frequency pulses of LH can lead to rapid regression of luteal cysts, restoring the normal function of the hypothalamic–pituitary-ovarian axis (26). While, compared to PGF2unction of the hypothalamic-pluteal cysts is higher than that of GnRH. Our research shows that no significant difference in the incidence of luteal cysts between the two groups was observed. The possible reason is that in PS, the injection scheme in which a first injection of PGF2halamo-pituitary-gonadal axis, by endogenous and/or exogenous factors, causes cysts formation and lack of statistical incidence of corpus luteal cysts. In DO, the applied Ovsynch procedure involved injecting PGF2that in PS, the injection scheme in which a first injection of PGF2halamo-pituitary-gonadal axis, by endogenous and/or exogenous factors, causes cyst formation and an effective treatment for this type of cysts (19). This may be the main reason for the lack of difference in the incidence of ovarian cysts between the two groups.

Follicular cysts are an early form of luteal cysts, and as they resolve or are treated, they may gradually undergo luteinization (27, 28). GnRH can promote a surge in luteinizing hormone (LH), which promotes the luteinization of follicular cysts. After 9 days, PGF2α is given to ablate the corpus luteum. The incidence of follicular cysts (29). Therefore, hormones that can induce the anterior pituitary gland to release LH, or have LH-like action (hCG), or LH itself can be used to treat follicular cysts (24, 29, 30). The combination of the progesterone-releasing device CIDR with Ovsynch demonstrated positive therapeutic effects on follicular cysts (19, 31). A single injection of progesterone can effectively reduce the lifespan of follicular cysts and increase the probability of ovulation (32). The reason is that the CIDR reduced and maintained LH secretion at normal luteum levels, thereby, inducing atresia of estrogen-active cysts and preventing formation of cysts from the newly emerged follicles (33). Moreover, administering GnRH before Ovsynch in cows with follicular cysts improved the pregnancy rates compared to Ovsynch alone; although this finding is still debated (34). Notwithstanding the low sensitivity of follicular cysts to PGF2α, the Ovsynch protocol had a therapeutic effect on them (35). This observation might explain the significantly lower incidence of follicular cysts in DO compared to PS in this study.

Interestingly, DO had a significantly lower incidence of inactive ovaries compared to PS. The pathogenesis of inactive ovaries is not clear, and previous reports have suggested a direct correlation with endometritis, retained placenta, twin pregnancies, and high fecundity, while an inverse correlation with parity (5, 36). In general, hormone therapy with a single injection of eCG or GnRH is commonly employed to restore ovarian function enabling the resumption of ovarian cycles within approximately 7 days. Although eCG is generally more effective than GnRH, it carries a higher risk of releasing twin eggs (13). Progesterone can increase blood flow to the uterus and follicles, thereby stimulating the ovaries to resume their activity cycle (37). Besides hormone therapy, cow-derived platelet-rich plasma (PRP) has demonstrated therapeutic effects on inactive ovaries, albeit with a longer duration (16, 22). In actual fact, following a 4-week injection of PRP into the ovaries, increased progesterone levels can be observed (22). In the current study, the incidence of ovarian inactivity was markedly lower in DO compared to PS. Only one cow in the DO group was diagnosed with an inactive ovary and cured, and could not be compared with the PS group.

There was no significant difference in the overall incidence of ovarian diseases between the DO and PS groups. In previous reports, the incidence rate of ovarian cysts detected through rectal examination ranged from 6 to 30% (4, 38). When hormone and B-mode ultrasound were used in combination for diagnosis, the incidence rate was between 18 and 29% (39, 40). The incidence rate of inactive ovaries is generally around 4.7 to 8.5% (5, 41). From this, it can be observed that the overall incidence rate of ovarian diseases and the incidence rates of different types of ovarian diseases in the DO group and PS group are lower than those reported in previous studies.

In this study, the conception rate of Double-Ovsynch was notably higher compared to Pre-Ovsynch, although the difference failed to reach statistical significance. Previous reports have demonstrated that double synchronization significantly enhances the first postpartum pregnancy rate compared to pre-synchronization, particularly for heifers and first-calving cows (42). However, no significant difference was observed in multiparous cows. This suggests that compared with PS, DO can better promote the recovery of ovarian cycle in postpartum cattle. Our results indicating a higher conception rate after recovery in DO compared to PS. This may also be one of the reasons why the pregnancy rate in the DO group is higher. However, these evidences are not sufficient, and more experimental data are needed to confirm this idea.

This experiment shows that compared with the PS protocol, the DO protocol can effectively reduce the incidence of follicular cysts and inactive ovaries, but has no effect on the incidence of luteal cysts. In addition, cows with treated ovarian diseases have a higher pregnancy rate after using the DO protocol, which may be one of the reasons for the higher pregnancy rate of the DO protocol. However, more experiments are needed to confirm this.

## Data availability statement

The original contributions presented in the study are included in the article/supplementary material, further inquiries can be directed to the corresponding author.

## Ethics statement

The animal studies were approved by the animal study was reviewed and approved by the Experiment Centre of Northwest A&F University. The studies were conducted in accordance with the local legislation and institutional requirements. Written informed consent was obtained from the owners for the participation of their animals in this study.

## Author contributions

ZiL: Conceptualization, Formal analysis, Investigation, Methodology, Writing – original draft, Validation. SL: Investigation, Methodology, Writing – original draft. LY: Data curation, Investigation, Writing – original draft. CX: Formal analysis, Validation, Writing – original draft. ZhL: Data curation, Writing – original draft. MY: Investigation, Writing – original draft. MM: Data curation, Writing – review & editing. PL: Methodology, Project administration, Writing – review & editing. AW: Supervision, Writing – review & editing. YJ: Funding acquisition, Resources, Supervision, Visualization, Writing – review & editing.
